# Recurrent Hyponatremia With Central Endocrine Dysfunction: A Diagnostic Challenge

**DOI:** 10.7759/cureus.104126

**Published:** 2026-02-23

**Authors:** Robert Li, Harpreet Sidhu, Jennifer Y Han

**Affiliations:** 1 Endocrinology, Diabetes and Metabolism, University of California Los Angeles David Geffen School of Medicine, Los Angeles, USA; 2 Nephrology, University of California Los Angeles David Geffen School of Medicine, Los Angeles, USA

**Keywords:** central hypothyroidism, hypogonadotropic hypogonadism, hyponatremia, hypopituitarism, pituitary cyst, siadh

## Abstract

A 55-year-old man with a history of chronic hyponatremia presented with recurrent nausea, vomiting, and severe hyponatremia. Initial evaluation revealed features consistent with the syndrome of inappropriate antidiuretic hormone secretion (SIADH), including hypotonic hyponatremia with inappropriately concentrated urine. Further workup demonstrated concurrent central hypothyroidism and hypogonadotropic hypogonadism, prompting neuroimaging. MRI identified a 1.7 cm suprasellar cystic mass compressing the pituitary infundibulum. The patient underwent transsphenoidal cyst drainage, resulting in the resolution of hyponatremia and normalization of pituitary function. This case highlights the importance of evaluating structural pituitary pathology in patients with hyponatremia and multiple endocrine abnormalities, demonstrating how compressive lesions can mimic idiopathic SIADH. The rapid postoperative normalization of sodium and hormonal axes underscores the reversibility of such deficits with timely intervention.

## Introduction

Hyponatremia, a common electrolyte disorder, has a multifactorial etiology that often presents diagnostic challenges [1]. The syndrome of inappropriate antidiuretic hormone secretion (SIADH) is a frequent cause of hyponatremia and is characterized by impaired free water excretion due to inappropriate vasopressin secretion [2]. When hyponatremia occurs together with other endocrine abnormalities, particularly pituitary hormone deficiencies, clinicians should be suspicious of central causes involving the hypothalamic-pituitary axis [3].

The pituitary gland's location and complexity make it vulnerable to mass effects from tumors, cysts, or other structural lesions [4]. Such lesions may disrupt normal hypothalamic-pituitary signaling and lead to specific patterns of hormonal dysfunction [5], including central hypothyroidism, characterized by low free thyroxine with inappropriately low or normal thyroid-stimulating hormone (TSH), and hypogonadotropic hypogonadism, characterized by low sex steroids with inappropriately low or normal gonadotropins [6].

Diagnosis and treatment of patients with hyponatremia and evidence of pituitary dysfunction requires integration of clinical presentation, biochemical testing, and neuroimaging findings [7]. Though SIADH may initially appear as an isolated disorder, the presence of additional endocrine abnormalities should prompt evaluation for structural pituitary lesions [8]. This clinical scenario underscores the importance of considering pituitary pathology in the differential diagnosis of persistent hyponatremia, particularly when it occurs in association with other hormonal deficiencies.

## Case presentation

A 55-year-old man with a medical history significant for chronic hyponatremia, gout, hypogonadism, and hypothyroidism presented with nausea, vomiting, acid reflux, and severe hyponatremia (sodium 118 mmol/L). Laboratory testing on admission (Table 1) revealed low serum osmolality (239 mOsm/kg) with inappropriately elevated urine sodium (134 mmol/L) and urine osmolality (488 mOsm/kg), which is consistent with SIADH.

**Table 1:** Admission labs showing hyponatremia, low serum osmolality, and inappropriately normal urine sodium concentration consistent with SIADH (LL): Data is critically low. (L): Data is abnormally low. SIADH: Syndrome of inappropriate antidiuretic hormone.

	Latest Reference Range and Units	May, 22, 2024, 16:56	May 23, 2024, 02:01	May 23, 2024, 09:51	May 23, 2024, 10:21
Na	134-146 mmol/L	118 (LL)	118 (LL)	116 (LL)	-
Osmolality, Serum	280-300 mOsm/kg	239 (LL)	-	-	-
Sodium, Urine Random	Not established mmol/L	-	-	-	134
Osmo Urine	500-800 mOsm/kg	-	-	-	488 (L)

The pattern of laboratory abnormalities mirrored a prior hospitalization a year earlier (Table 2), where he was similarly diagnosed with SIADH and treated with two liters per day fluid restriction and 2 g per day NaCl tablets.

**Table 2:** Electrolyte abnormalities on the previous admission showing hyponatremia, low serum osmolality, and inappropriately normal urine sodium concentration, consistent with SIADH (L) Data is abnormally low.

	Latest Reference Range and Units	September 13, 2023, 04:49	September 13, 2023, 13:01	September 13, 2023, 13:23	September 13, 2023, 13:27	September 14, 2023, 04:29
Na	134-146 mmol/L	127 (L)	128 (L)	-	-	128 (L)
Osmolality, Serum	280-300 mOsm/kg	260 (L)	-	-	-	-
Sodium, Urine Random	Not established mmol/L	-	-	-	81	-
Osmolality, Urine	500-800 mOsm/kg	-	-	317 (L)	-	-

Further evaluation revealed a history of long-standing central hypothyroidism, with low thyroid-stimulating hormone (TSH) and free T4 (Table 3), and hypogonadotropic hypogonadism (Table 4), with testosterone levels as low as <3 ng/dL and inappropriately normal gonadotropin (luteinizing hormone (LH), follicle-stimulating hormone (FSH)) levels.

**Table 3:** Concurrently low thyroid-stimulating hormone (TSH) and free T4 levels on multiple admissions consistent with central hypothyroidism (L) Data is abnormally low.

		January 6, 2023	September 13, 2023	February 23, 2024	May 17, 2024	May 22, 2024	May 23, 2024	Reference Range and Units
TSH	1.52		0.497 (L)	1.151	0.387 (L)	0.5 (L)	0.33 (L)	0.550-4.780 uIU/mL
Free T4		-	0.58 (L)	0.92	-	0.74 (L)	0.66 (L)	0.89-1.76 ng/dL

**Table 4:** History of low serum testosterone with inappropriately normal gonadotropins consistent with central hypgonadism (L) Data is abnormally low.

	January 6, 2023	September 1, 2023	September 13, 2023	November 8, 2023	February 23, 2024	Reference Range
Testosterone	177 (L)	<3 (L)	38 (L)	141.9 (L)	177 (L)	264-916 ng/dL
Luteinizing hormone (LH)	-	6.5	-	-	-	1.8-8.6 mIU/mL
Follicle-stimulating hormone (FSH)	-	9	-	-	-	1.5-12.4 mIU/mL

**Figure 1:**
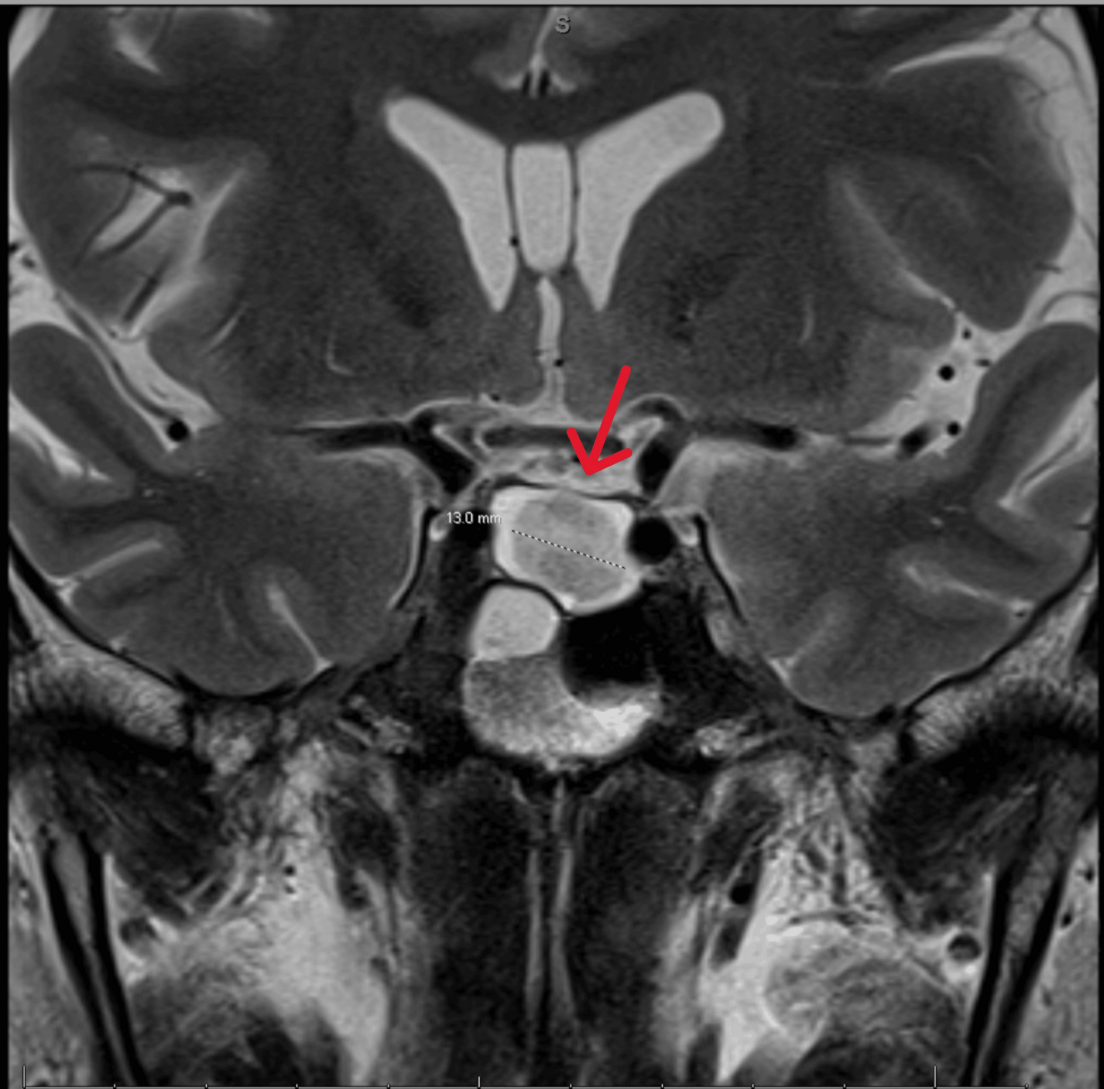
MRI pituitary with and without contrast showing a suprasellar mass in the posterior pituitary Posterior pituitary mass (red arrow) measuring 1.6x1.6x1.7 cm with displacement of the infundibulum and anterior pituitary gland. There is a mass effect on the infundibulum. Differential diagnosis includes craniopharyngioma, neurosarcoidosis versus pituitary apoplexy.

The combination of central hypothyroidism, central hypogonadism, and SIADH raised suspicion of a pituitary etiology. MRI pituitary with and without contrast (Figure 1) identified a 1.7 cm suprasellar mass in the posterior pituitary, exhibiting T1 hyperintensity, suggestive of proteinaceous or hemorrhagic content. The mass caused anterior displacement of the infundibulum and the anterior pituitary, which likely accounted for the patient’s hormonal abnormalities.

Neurosurgery was consulted and the neurosurgery team performed a trans-nasal trans-sphenoidal (TNTS) drainage of the suprasellar cyst. Postoperatively, the patient’s hyponatremia resolved and endocrine function normalized. Follow-up laboratory testing (Table 5) four months later showed normalization of sodium levels, free T4, TSH, and testosterone, confirming restoration of pituitary-hypothalamic axis function.

**Table 5:** Post-operative labs showing normalization of all pituitary hormones and all electrolytes

Test	October 21, 2024	Reference Range
Sodium	141 mmol/L	136-145 mmol/L
Potassium	4.3 mmol/L	3.5-5.1 mmol/L
Chloride	108 mmol/L	98-107 mmol/L
CO_2_	26 mmol/L	22-29 mmol/L
Cortisol	7.2 mcg/dL	No Ref Range
Free T4	1.09 ng/dL	0.7-1.48 ng/dL
Thyroid-stimulating hormone (TSH)	0.560 μIU/mL	0.350-4.940 μIU/mL
Luteinizing Hormone (LH)	4.41 mIU/mL	No Ref Range
Prolactin	7.80 ng/mL	3.46-19.40 ng/mL
Testosterone Total	371 ng/dL	240-950 ng/dL

## Discussion

This case demonstrates important aspects of hyponatremia and pituitary dysfunction. The patient's chronic hyponatremia, initially diagnosed as SIADH, was ultimately found to be secondary to mass effect from a pituitary mass compressing the infundibulum (pituitary stalk).

Pituitary lesions compressing the infundibulum can cause hyponatremia through several mechanisms, as given below:

*SIADH-like mechanism*:* *Inappropriate ADH secretion as a result of compression of the infundibulum can impair normal inhibitory signals to the posterior pituitary [9]. This form is often termed "secondary SIADH" because it results from structural interference with hypothalamic-pituitary signaling [8]. Key features include impaired osmotic suppression of antidiuretic hormone (ADH) [10] and euvolemic hyponatremia with inappropriately concentrated urine (urine osmolality >100 mOsm/kg despite low serum osmolality) [2].

*Secondary adrenal insufficiency (cortisol deficiency)*:* *Disruption of adrenocorticotropic hormone (ACTH) secretion from compression of the anterior pituitary or infundibulum can lead to glucocorticoid deficiency [4]. Since cortisol normally inhibits ADH release, its deficiency can result in hyponatremia from uninhibited ADH secretion [11] as well as impaired free water excretion [12].

*Combined anterior and posterior pituitary dysfunction*:* *Pituitary masses can in some cases cause a triphasic response (often seen after pituitary surgery or trauma): (a) initial ADH surge (acute hyponatremia due to excessive ADH release from injured neurons); (b) diabetes insipidus (DI) (if ADH-secreting neurons are destroyed); (c) chronic SIADH (if aberrant ADH release resumes from damaged axons) [9].

Differentiating SIADH from adrenal insufficiency is critical to the evaluation of hyponatremia. This can be done by measuring a morning cortisol. A morning cortisol <3 µg/dL suggests adrenal insufficiency, while normal or high cortisol suggests SIADH-like physiology [3]. If a pituitary lesion is suspected, an MRI is used as a diagnostic tool [5].

Treatment depends on the mechanism of hyponatremia. If the etiology of the hyponatremia is SIADH-like, fluid restriction, salt tablets, or vaptans are the preferred treatment [8]. If the hyponatremia arises from adrenal insufficiency, glucocorticoid replacement is needed [4]. Definitive management involving surgical cyst drainage or adenoma removal often corrects both the hyponatremia and any pituitary hormonal deficits [7].

In this case, the concurrent presence of central hypothyroidism and hypogonadotropic hypogonadism along with an SIADH clinical picture strongly suggested hypothalamic-pituitary dysfunction [7]. The patient's hyponatremia and pituitary hormone deficiencies completely resolved following surgical drainage of the pituitary cyst, which confirmed the diagnosis of the pituitary cyst causing secondary SIADH and hypopituitarism. The successful resolution of both hyponatremia and hormonal deficiencies after surgical intervention underscores the importance of treating the underlying pathology in such cases [7]. While hormone replacement can be used to manage symptoms and SIADH can be treated with fluid restriction and salt replacement, accurate diagnosis and definitive treatment may completely reverse the endocrine abnormalities, as seen in this patient.

## Conclusions

This case emphasizes the importance of a comprehensive endocrine evaluation including early and appropriate neuroimaging in patients with persistent hyponatremia. When multiple hormonal abnormalities coexist, biochemical and radiological workup for pituitary pathology needs to be performed. With appropriate surgical intervention of the underlying pituitary pathology, a potential for complete recovery is possible.

## References

[1] Adrogué HJ, Madias NE (2000). Hyponatremia. N Engl J Med.

[2] Ellison DH, Berl T (2007). Clinical practice. The syndrome of inappropriate antidiuresis. N Engl J Med.

[3] Fenske W, Allolio B (2012). Clinical review: current state and future perspectives in the diagnosis of diabetes insipidus: a clinical review. J Clin Endocrinol Metab.

[4] Fleseriu M, Hashim IA, Karavitaki N, Melmed S, Murad MH, Salvatori R, Samuels MH (2016). Hormonal replacement in hypopituitarism in adults: an Endocrine Society Clinical Practice Guideline. J Clin Endocrinol Metab.

[5] Freda PU, Beckers AM, Katznelson L, Molitch ME, Montori VM, Post KD, Vance ML (2011). Pituitary incidentaloma: an endocrine society clinical practice guideline. J Clin Endocrinol Metab.

[6] Higham Higham, CE CE, Johannsson Johannsson, G G, Shalet Shalet, SM SM (2016). Hypopituitarism. Lancet.

[7] Melmed S (2020). Pituitary-tumor endocrinopathies. N Engl J Med.

[8] Verbalis JG, Goldsmith SR, Greenberg A, Korzelius C, Schrier RW, Sterns RH, Thompson CJ (2013). Diagnosis, evaluation, and treatment of hyponatremia: expert panel recommendations. Am J Med.

[9] Olson BR, Gumowski J, Rubino D, Oldfield EH (1997). Pathophysiology of hyponatremia after transsphenoidal pituitary surgery. J Neurosurg.

[10] Verbalis JG (2014). Hyponatremia with intracranial disease: not often cerebral salt wasting. J Clin Endocrinol Metab.

[11] Oelkers W (1989). Hyponatremia and inappropriate secretion of vasopressin (antidiuretic hormone) in patients with hypopituitarism. N Engl J Med.

[12] Hannon MJ, Thompson CJ (2019). Hyponatremia in neurosurgical patients. Front Horm Res.

